# PreDBA: A heterogeneous ensemble approach for predicting protein-DNA binding affinity

**DOI:** 10.1038/s41598-020-57778-1

**Published:** 2020-01-28

**Authors:** Wenyi Yang, Lei Deng

**Affiliations:** 1 School of Computer Science and Engineering, Central South University, Changsha, 410075 China; 2 School of Software, Xinjiang University, Urumqi, 830008 China

**Keywords:** Computational models, Machine learning

## Abstract

The interaction between protein and DNA plays an essential function in various critical natural processes, like DNA replication, transcription, splicing, and repair. Studying the binding affinity of proteins to DNA helps to understand the recognition mechanism of protein-DNA complexes. Since there are still many limitations on the protein-DNA binding affinity data measured by experiments, accurate and reliable calculation methods are necessarily required. So we put forward a computational approach in this paper, called PreDBA, that can forecast protein-DNA binding affinity effectively by using heterogeneous ensemble models. One hundred protein-DNA complexes are manually collected from the related literature as a data set for protein-DNA binding affinity. Then, 52 sequence and structural features are obtained. Based on this, the correlation between these 52 characteristics and protein-DNA binding affinity is calculated. Furthermore, we found that the protein-DNA binding affinity is affected by the DNA molecule structure of the compound. We classify all protein-DNA compounds into five classifications based on the DNA structure related to the proteins that make up the protein-DNA complexes. In each group, a stacked heterogeneous ensemble model is constructed based on the obtained features. In the end, based on the binding affinity data set, we used the leave-one-out cross-validation to evaluate the proposed method comprehensively. In the five categories, the Pearson correlation coefficient values of our recommended method range from 0.735 to 0.926. We have demonstrated the advantages of the proposed method compared to other machine learning methods and currently existing protein-DNA binding affinity prediction approach.

## Introduction

The interaction between protein and DNA is one of the kernel problems in molecular biology and plays significant roles in several biological actions, such as DNA replication, repair, and alteration processes^1^. Researchers have been focused on analyzing the interactions of proteins to DNA^2–4^ to understand the identification mechanism of protein-DNA complexes. During the past few years, many laboratory programs for investigating protein binding have been proposed. Electrophoretic mobility shift assays (EMSAs)^5,6^, conventional chromatin immunoprecipitation (ChIP)^7^, peptide nucleic acid (PNA) assisted identification of RNA-binding proteins (RBPs) (PAIR)^8^, X-ray crystals^9^ and nuclear magnetic resonance (NMR) spectroscopy^10^ have been applied to expose protein-DNA binding residues. However, these laboratory methods are expensive and time-consuming. Alternatively, low cost and efficient computational methods are particularly meaningful toward studying the interaction of protein-DNA complexes.

Quantitative prediction of protein-DNA binding affinity is essential for the recognition of protein-DNA interactions. Many computational prediction techniques, including empirical scoring functions^11–15^, knowledge-based methods^16–18^ and quantitative structure-activity relationships^19,20^, have been proposed for the binding affinity of protein-ligand complexes and protein-protein complexes^21–23^. Although there have been many methods to develop the scoring functions in protein-ligand and protein-protein docking simulations, most of them are based on a series of binding affinities benchmarks^24,25^. However, this is a requirement for growing and establishing protein-DNA binding affinity.

**Figure 1:**
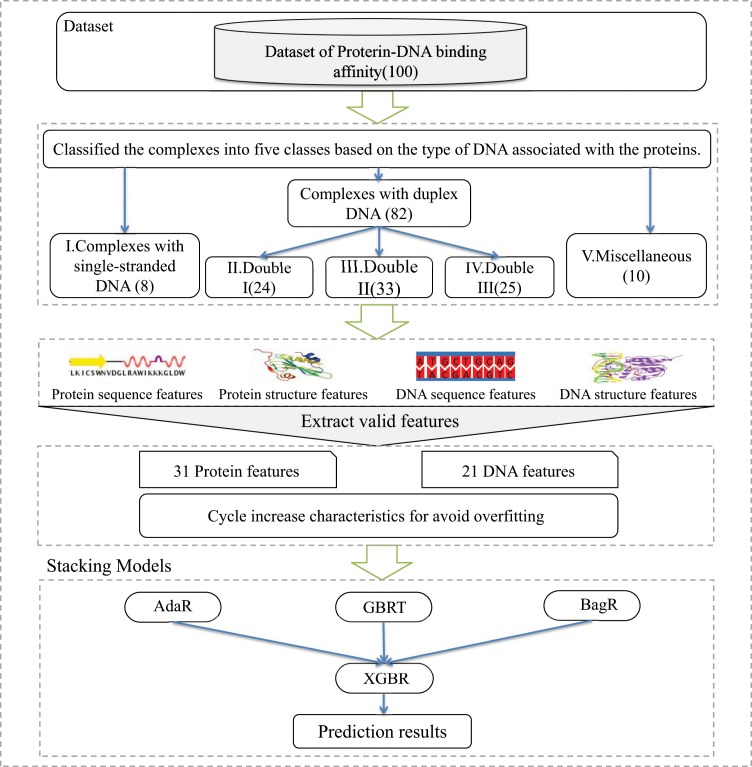
The flowchart of PreDBA. The main four steps: (**A**) Extract protein-DNA complexes from the literature; (**B**) Classify the protein-DNA complexes by the type of DNA associated with the protein; (**C**) Extract valid sequence and structural features; (**D**) Build stacking heterogeneous ensemble models.

In this paper, a novel computational method named PreDBA is proposed to predict the protein-DNA binding affinity quantitatively. Figure 1 shows the flowchart of our way. According to the style of DNA that interacts with protein^26^, we classify the protein-DNA complexes into five groups. For each class, a heterogeneous ensemble model is constructed to predict the binding affinity. For each class of the protein-DNA complex, we performed a systematic analysis of whether the features affect predicted binding affinity. The results show that structural features are significant for controlling protein-DNA binding affinity. The Pearson correlation coefficient of our method based on the cross-validation of the leave-one-out^27^ method reached 0.735 to 0.926. Moreover, the results show that our approach is superior to several classic regression methods and popular binding affinity prediction methods. Besides, we have developed a user-friendly webserver to predict the binding affinity of protein-DNA complexes.

## Results

### Datasets

We manually curated a set of 201 protein-DNA complexes with experimentally determined binding affinity from the literature. We only selected the protein-DNA crystal structures deposited in the PDB that have better than 3 resolution. Proteins with sequence similarity >40 % were excluded by using CD-HIT^28^. At last, we got 100 protein-DNA complexes and built the binding affinity dataset (displayed in the Supplementary Table) along with the laboratory conditions (temperature). Dissociation Gibbs free energy (Δ*G*) is used to measure the binding affinity^21^, which is calculated as follows:

1 $$\Delta G=-RT\ {\rm{ln}}\ {K}_{d},$$

where T is the temperature, R is the gas constant (1.987 × 10^−3^kcal mol^−1^ K^−1^), and K_*d*_ is the dissociation constant.

### Classification of complexes

It is deserving noting that previous studies have illustrated that the interaction between proteins and DNAs^2^ is associated with the structure of the DNA molecule, that is, various features related to the construction of DNA will affect the binding affinity of various class of DNA. Previous studies have built predictive models^2^ by classifying protein-DNA complexes by different kinds of DNA. Therefore, based on the rule of the Nucleic Acid Database (NDB)^26^, the protein-DNA complexes are divided into three categories: I) complexes with single-stranded DNA (SS), II) complexes with duplex DNA, III) miscellaneous complexes (MISC).

According to previous studies^29,30^, it has been confirmed that protein-DNA binding site residues have an essential influence on the interaction of protein and DNA. Actually, the binding site residues are believed to play essential roles in directing the binding affinity. To balance the amount of each class of the protein-DNA complexes, we further divided the compounds with duplex DNA into three various categories based on the percentage of binding site residues in the protein of the protein-DNA complexes according to previous research^21^, viz., Double I, Double II and Double III (≤10%, 10–20% and ≥20% of binding site residues, respectively). Some guidelines have been proposed to identify the DNA-binding sites in previous research, such as the distance between contacting atoms in protein and DNA^31^, reduction in solvent accessibility on binding^32^ and interaction energy between protein and DNA^33^. The distance-based criteria are used in most of the prediction studies for analyzing the binding sites of protein-DNA complex to identify binding sites. In our work, a residue in the DNA-binding protein is defined as a binding site if the distance between any protein atoms and DNA atoms is ≤5.0.

### Regression models and performance evaluation

We train the stacking heterogeneous ensemble method using the selected features for every class of protein-DNA complexes to predict binding affinities. First, we use three different regression methods to create predictions (Adaboost Regression (AdaR)^34^, Gradient Boosted Regression Tree (GBRT)^35^ and Bagging Regression (BagR)^36^), then we integrate them up by XGBoost Regression (XGBR)^37^ to make a terminal forecast.

We used Pearson’s correlation coefficient^38^ to assess the correlation between the predicted values and experimental values. Moreover,the Pearson correlation coefficient *r* is defined as follows:

2 $$r=\frac{{\sum }_{i=1}^{n}({a}_{i}-\bar{a})({b}_{i}-\bar{b})}{\sqrt{{\sum }_{i=1}^{n}{({a}_{i}-\bar{a})}^{2}}\sqrt{{\sum }_{i=1}^{n}{({b}_{i}-\bar{b})}^{2}}},$$

where *n* represents the number of samples, *a*_*i*_, *b*_*i*_ are the ith sample, and $\bar{a}$ and $\bar{b}$ represent the mean of the samples, i.e. $\bar{a}=\frac{1}{n}{\sum }_{i=1}^{n}{a}_{i}$ and $\bar{b}=\frac{1}{n}{\sum }_{i=1}^{n}{b}_{i}$.

Besides, both the mean absolute error (MAE)^39^ and the determination coefficient (R2)^40^ can be used to assess the relationship between predicted and actual values.

### Significance of different classifications

To verify the significance of DNA type for protein-DNA complexes classification, we performed the following experiments. Instead of using all the complexes as a whole, one and two optional characteristics are applyed to train the prediction model, respectively. We use one and two optional characteristics for each class to build a heterogeneous ensemble prediction models and calculate the performance indicators separately. As can be seen from Table 1, the prediction accuracy after classifying the complexes is much better than the prediction accuracy before classification.

**Table 1:** Correlation results for different classifications with the optional one and two features.

Class	Number of complexes	Maximum correlation coefficient(r)	
		Single property	Two properties
SS	8	0.513	0.762
Double I	24	0.466	0.632
Double II	33	0.474	0.562
Double III	25	0.482	0.643
MISC	10	0.502	0.693
Together	100	0.165	0.443

**Figure 2:**
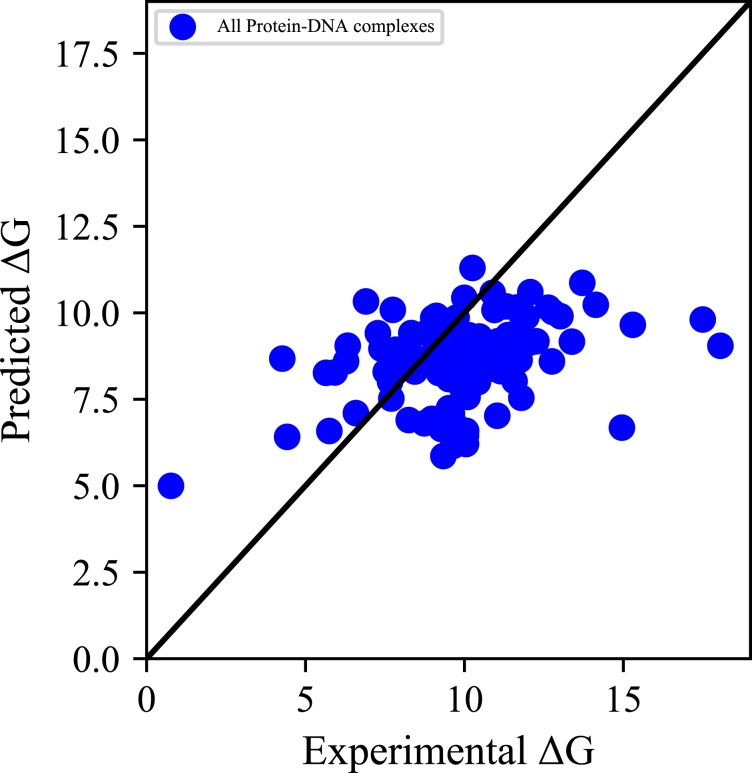
Scatterplot of predicted binding affinities of protein-DNA complexes before classification.

**Figure 3:**
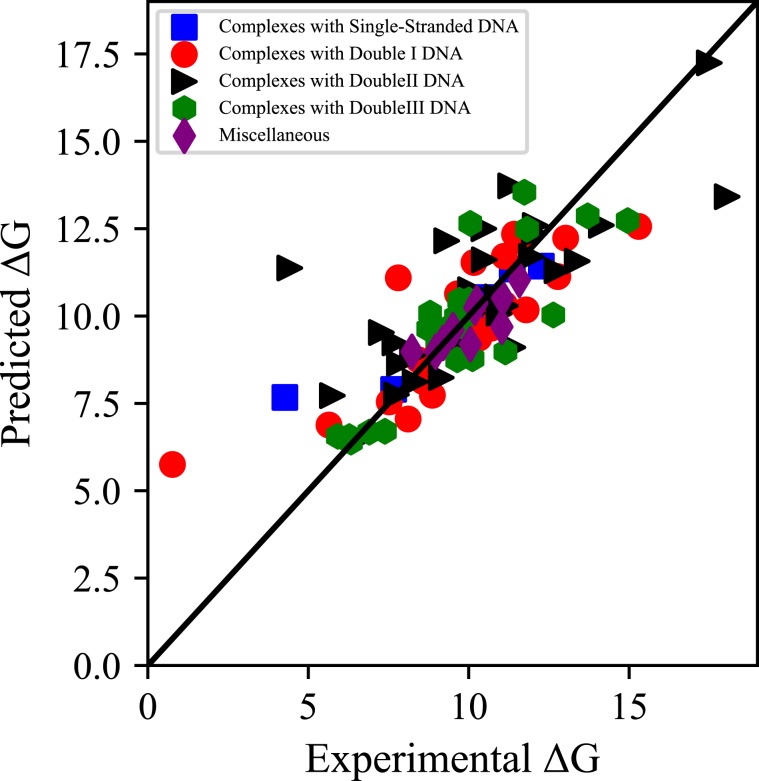
Scatterplot of predicted binding affinities of protein-DNA complexes after classification.

In all five groups of complexes, the correlation coefficient of a predictive model based on an optional feature is higher than 0.45. But the entire complexes have a correlation coefficient of only 0.165. And the two properties correlation coefficient is > 0.5 in all of the types. Moreover, the scatter plot of the experimental *vs* predicted binding affinity are shown in Figs. 2 and 3. Figures 2 and 3 shows the experimental and predicted Δ*G* of all the protein-DNA complexes before and after classification, respectively. As can be seen from Fig. 3, most points positioned close to the diagonal line. And at the same time, most of the points in Fig. 2 are randomly distributed. Pre- and post-classification comparisons illustrate that our approach of using classification before predicting the protein-DNA binding affinity is effectual. The reason for the difficulty in modeling may be the weak correlation between different classes of complexes. Therefore, before establishing a practical predictive model, the importance of the classification of the protein-DNA complexes are stressed.

### Prediction of binding affinity

We established regression models for each protein-DNA complexes to do the prediction of the protein-DNA binding affinity. The performance of our method are displayed in Table 2. The Pearson’s correlation coefficients for all complex categories are greater than 0.73, which means that the predicted binding affinity is closely related to the actual value. Moreover, the great value of other evaluation criteria also prove the superiority of our approach. All results of the performance evaluation measures have proved our method is useful, and the classification can improve the accuracy of the algorithm effectively.

**Table 2:** Performance of PreDBA by using leave-one-out cross-validations.

	Correlation coefficient(r)	Mean absolute error(MAE)	Coefficient of determination(R2)	Running time
SS	0.940	0.639	0.713	0.55s
Double I	0.829	1.172	0.667	3.34s
Double II	0.752	1.234	0.557	4.41s
Double III	0.843	0.882	0.700	2.04s
MISC	0.834	0.433	0.622	0.26s
Average value	0.840	0.872	0.652	2.12s

**Figure 4:**
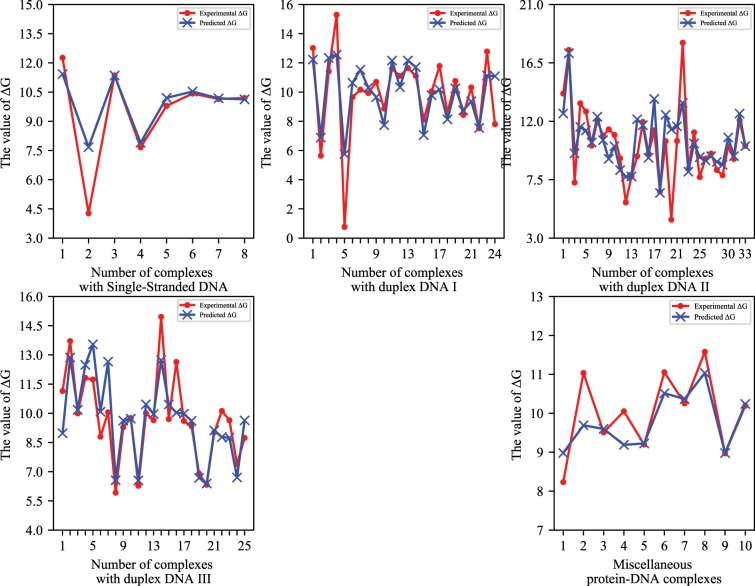
The predicted and real binding affinities of every complex among five classes.

Next, to further explore the characteristics of the governing protein-DNA complex binding affinity prediction, we evaluate the prediction performance of the methods in various classes. Figure 4 shows the predicted and actual values of the binding affinity of each of the five types of complexes, respectively. As shown in Fig. 4, we can see that, except for a few individual positions, most predicted binding affinity values closely match the corresponding experimental binding affinity values for each protein-DNA complex. We have analyzed the performance of the approach we used to predict the binding affinity for each group of protein-DNA complexes, and the details are described below.

#### Complexes with single-stranded DNA

For this class, the protein fraction of the complex binds to single-stranded DNA. There are eight protein-DNA complexes in this group, with a smallest binding affinity of 4.3 kcal mol^−1^, with a varied range of 8 kcal mol^−1^, up to 12.3 kcal mol^−1^. Based on these 4 various characteristics, the Pearson’s correlation coefficient of our model reached 0.94 by using leave-one-out cross-validation method. Further, the mass of the beta sheet has been identified as the essential factor of predicting the protein-DNA binding affinity. Moreover, the number of the beta sheet of the protein and the pairwise interactions GA/CT and GC/CG have also played a vital role in protein binding to DNA. As can be seen from our predictions, our approach could accurately predict the binding affinity of 87.5% of the complexes with a deviation of 1 kcal mol^−1^ using the leave-one-out test.

#### Complexes with duplex DNA

This type of protein-DNA complex includes two parts: protein and double-stranded DNA. We have divided this type of complex into three categories, namely Double I, Double II, and Double III. Below we will introduce which features affect these types of compounds. The specific prediction results for each type of complex are displayed below.

1. Double I. There are 24 complexes in this class, and we can find the absolute value of the binding free energy is at least 0.767, and the maximum is 15.298 kcal mol^−1^. Through our model prediction, we get a correlation coefficient of 0.829. The percentage of the polar residues in the protein played a decisive role in predicting the results among all the characteristics we obtained. The number of Watson-Crick base-pairs XIX and the portion of Watson-Crick base-pairs XIX play a proper position in predicting protein-DNA binding affinity. The binding affinity for 20 and 18 of 24 complexes has been accurately predicted within the deviation of 2 and 1 kcal mol^−1^, respectively, using the leave-one-out test.
2. Double II. Double II has 33 complex samples, which is the category with the most significant number of samples in all classes. Four chosen characteristics are applied to develop the forecast model that obtains the correlation coefficient 0.752. In our prediction process, the mass of the alpha helix and the number of hydrophilic residues in the protein played a decisive role in our results based on the characteristics of the protein fraction. Regarding DNA-based features, the Nearest-neighbor bases of DNA play a crucial role. The pairwise interactions AA/TT, CA/GT are essential for the prediction. The binding affinity for 28 of 33 complexes has been accurately predicted within the deviation of 2 kcal mol^−1^ using the leave-one-out test.
3. Double III. Double III is a collection of the binding sites of protein-DNA more than 20% in protein-binding double-stranded DNA, with 25 complex samples. And the absolute average value of ΔG is 9.7 kcal mol^−1^. Through the prediction of three characteristics, we can get a correlation coefficient of 0.843. In this class of complex binding affinity prediction process, we found that the Nearest-neighbor bases of DNA play a decisive role. The binding affinity for 20 and 17 of 24 complexes has been accurately predicted within the deviation of 2 and 1 kcal mol^−1^, respectively, using the leave-one-out test.

#### Miscellaneous complexes

The Miscellaneous has twenty complex samples, and the absolute average value of the binding free energy ΔG of this class is 10.01 kcal mol^−1^. For this class of complexes, we used four features to built the forest model for the prediction of the protein-DNA binding affinity and obtained a correlation coefficient of 0.834. We found that the protein aspect that plays a decisive role in predicting the results. The molecular mass and the amount of the alpha helix in protein are two meaningful features. Meanwhile, the amount of aromatic and positively charged residues in the protein and the total amount of hydrogen bonds in protein are all important for the prediction. By observing the prediction results, we found that the features we used have a beneficial effect on predicting the binding affinity of the miscellaneous. Our approach could precisely predict the binding affinity of 90% of the complexes with a deviation of 1 kcal mol^−1^ using the leave-one-out test.

### Comparison of PreDBA with other regression methods

**Figure 5:**
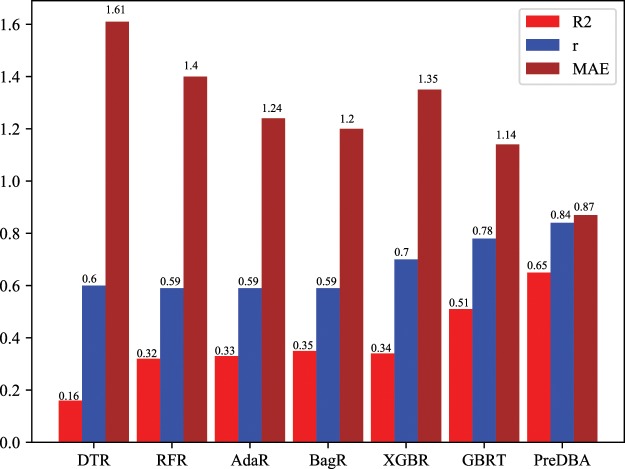
Comparison of mean performance evaluation measures over five classes of protein-DNA complexes between PreDBA and typical regression methods.

The performance of PreDBA can be evaluated by comparing it with the other six regression methods: Decision Tree Regression (DTR)^41^, Random Forest Regression (RFR)^42^, Adaboost Regression (AdaR), Bagging Regression (BagR), XGBoost Regression (XGBR), and Gradient Boosted Regression Tree (GBRT). As shown in the Table 3, the performance of PreDBA for all categories of complexes is significantly better than other regression models. In addition, we also calculated the average of the performance indicators of various regression models, as shown in Fig. 5. The average correlation coefficient of the PreDBA model reached 0.84, and the average MAE value equal to 0.88, and the average R2 value is 0.65, which are higher than the other four methods. It is conclude that the heterogeneous ensemble model makes our approach perform better than other regression methods.

**Table 3:** Comparison of the correlation coefficient of PreDBA with other regression algorithms.

	DTR	RFR	AdaR	BagR	XGBR	GBRT	PreDBA
SS	0.889	0.672	0.734	0.721	0.830	0.889	**0.940**
Double I	0.486	0.543	0.515	0.521	0.559	0.771	**0.829**
Double II	0.449	0.484	0.453	0.479	0.639	0.702	**0.752**
Double III	0.550	0.559	0.563	0.548	0.701	0.773	**0.843**
MISC	0.643	0.701	0.687	0.676	0.788	0.744	**0.834**
Average	0.60	0.59	0.59	0.59	0.70	0.78	**0.84**

In order to verify the validity of the machine learning algorithms utilized in our stacking model, we analyzed the effects of different algorithm combinations. Table 4 shows the correlation coefficient between predicted binding affinities and real values by using different algorithm combinations in the first layer of the stacking model. As can be seen from Table 4, different model combinations have various effects on the prediction results. The performance of our PreDBA method combining all the three algorithms (GBRT+AdaR+BagR) is better than using only one or two algorithms.

**Table 4:** Comparison of the correlation coefficient of PreDBA with other algorithm combinations in the stacking model.

	SS	Double I	Double II	Double III	MISC	Average
GBRT	0.872	0.792	0.711	0.801	0.762	0.79
AdaR	0.757	0.536	0.501	0.577	0.704	0.62
BagR	0.736	0.554	0.514	0.587	0.694	0.62
GBRT+AdaR	0.874	0.792	0.713	0.783	0.756	0.78
AdaR+BagR	0.772	0.634	0.652	0.602	0.732	0.68
GBRT+BagR	0.904	0.765	0.732	0.795	0.806	0.80
GBRT+AdaR+BagR(PreDBA)	0.940	0.829	0.752	0.843	0.834	0.84

### Comparison with state-of-the-art approach

As far as we know, there is only one existing protein-DNA binding affinity quantitative prediction method DDNA3^43^. DDNA3 is an upgraded version of DDNA^44^. DDNA3 uses a knowledge-based energy function to predict protein-DNA complex binding affinity. We apply DDNA3 to predict the binding affinity of the complexes by using our data set and contrast it to our method. Table 5 shows the comparison of the DDNA3 process with our approach by using the correlation coefficient criterion. From this table, we can see that our PreDBA is significantly better than DDNA3 in predicting each class.

**Table 5:** Comparison of correlation coefficients between DDNA3 and PreDBA.

Class	Correlation coefficient(r)	
	DDNA3	PredDBA
SS	0.703	0.940
Double I	0.738	0.829
Double II	0.688	0.752
Double III	0.742	0.843
MISC	0.756	0.834
Average	0.73	0.84

### Web server

We develop a web server to predict the protein-DNA binding affinity available to the research community, which is freely accessible at http://predba.denglab.org/. The PreDBA web server is developed in Perl, Python, JavaScript, jQuery (AJAX), and CSS. It accepts protein-DNA complex 3D structures in PDB format or PDB codes as input. The binding affinities of the protein-DNA complexes will be predicted and displayed.

## Discussion

In this paper, we generate a non-redundant dataset of protein-DNA binding affinity, which In this paper, we generate a non-redundant dataset that contains binding affinity values of one hundred protein-DNA complexes. Based on the structural classification, we developed a way termed PreDBA by using heterogeneous ensemble models to forecast the protein-DNA binding affinities. By using the leave-one-out cross-validation procedure,the mean correlation coefficient we obtained is 0.82. For understand the importance of selected features for protein-DNA binding affinity in each class, we systematically analyzed the features of all classes. We also compared the regression approach we used with some different standard regression methods and proved that our approach has the most significant effect. Furthermore, we compared PreDBA with the pioneer protein-DNA binding affinity prediction method DDNA3, and the results confirm that PreDBA does have a better outcome. Finally, we have developed a web server (http://predba.denglab.org/) that can be used to predict binding affinity of protein-DNA affinity freely. We hope our PreDBA method can be helpful for the study of all aspects of the interaction between protein and DNA.

## Methods

### Features extraction

We obtain 52 characteristics to forecast the binding affinity of the protein-DNA compounds. The characteristics are principally come from the structural and sequential information of proteins and DNA in the protein-DNA compounds. The specific characteristics are listed below.

#### Protein sequential features

The sequential information of protein are extracted from the PDB files. Based on each amino acid in the protein sequence, we then calculated the molecular mass^45^ of the protein sequence . Also, we assessed the whole amount of hydrogen bonds^46^ included in the protein sequence. Moreover, based on the sequence information of the protein, we calculated the physical and chemical properties, including the hydrophilic and hydrophobic residues^47^ in the protein, the aromatic and positively charged residues^48^ in the protein, the polar residues in the protein and the charged residues in the protein.

#### Protein structural features

The tool we applied to get the protein secondary structure information is the DSSP algorithm. The secondary structure of protein mainly including the amount and the portion of the alpha helix and the beta sheet in the protein, the molecular mass of the alpha helix^49,50^ and the beta sheet^51^. Meantime, the solvent-accessible surface area (SASA)^52^ of the protein are collected.

#### DNA sequential features

Based on DNA base sequential information, we obtained two features for predicting binding affinity, as described below.

1. DNA Molecular mass. We used the sequence information of the DNA in the complex to gain the molecular weight of the DNA sequence. The molecular mass of single-stranded DNA (ssDNA) and double-stranded DNA (dsDNA) are calculated as follows:
  3 $${W}_{ssDNA}=Nu{m}_{nucleotides}\ast 303.7+79.0$$
  4 $${W}_{dsDNA}=Nu{m}_{nucleotides}\ast 607.4+157.9$$
  where Num_*nucleotides*_ is the number of nucleotides, 303.7 and 607.4 represent the average molecular mass of bases of ssDNA and dsDNA, respectively.
2. DNA nearest-neighbor bases^53^. Ten different nearest-neighbor interactions are likely in each Watson-Crick DNA duplex structure. These pairwise interactions we used are CA/GT; GC/CG; GG/CC; CG/GC; TA/AT; AT/TA; AA/TT; GT/CA; GA/CT; CT/GA. We collected the amount of DNA nearest-neighbor bases.

#### DNA structural features

Some features based on DNA structure to predict protein-DNA binding affinity are shown below. The RNAfold tool in ViennaRNA2.4.3^54^ is used to forest the ensemble diversity and the frequency of the minimum free energy (MFE) structure. Also, the features of cWW (Cis Watson-Crick/Watson-Crick)^55^ are predicted. The 28 possible base-pairs^26^ for A, G, T, and C involving at least two (cyclic) hydrogen bonds. We get the number of Watson-Crick base-pairs XIX and XX and their percentage in the base sequence.

### Features selection

Since the binding affinities of different categories of compounds have a significant correlation with the structure of DNAs and proteins, we perform feature selection for each type of protein-DNA compound iteratively and independently. For each type of complex, we use correlation coefficients to measure the relationship between each feature and binding affinity. Next, the calculated correlation coefficients are sorted in descending order, and the top 10 features are selected for each type of complexes. Ultimately, the greedy algorithm are used to select the appropriate feature set for each type of complex until the capability no longer improves. Selected features of each protein-DNA complexes are shown in the Table 6. In general, to avoid overfitting, the final optimal feature set contains should less than five features for all five groups of complexes.

**Table 6:** Features selected in each class of complexes.

	SS	D I	D II	D III	MISC
the number of DNA nearest neighbor bases AA/TT			✓	✓	
the number of DNA nearest neighbor bases CA/GT			✓	✓	
the number of DNA nearest neighbor bases GA/CT	✓			✓	
the number of DNA nearest neighbor bases GG/CC					
the number of DNA nearest neighbor bases GC/CG	✓				
the number of the aromatic and positively charged residues in the protein					✓
% of the polar residues in protein		✓			
the amount of the alpha helix in protein					✓
the amount of the beta sheet in protein	✓				
the amount of Watson-Crick base-pairs XIX		✓			
% of Watson-Crick base-pairs XIX		✓			
the molecular mass of the alpha helix			✓		✓
the molecular mass of beta sheet	✓				
the amount of hydrophilic residues			✓		
the total number of hydrogen bonds of the protein fraction					✓

### The stacking heterogeneous ensemble method

**Figure 6:**
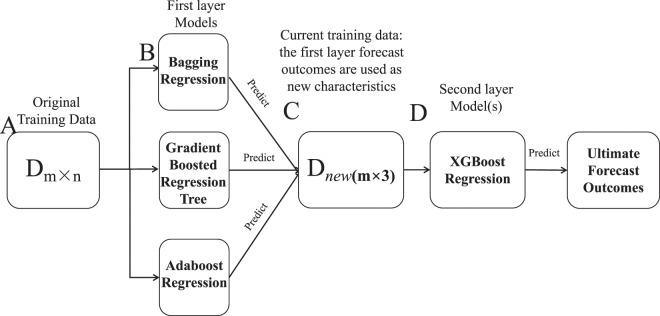
The flowchart of the PreDBA method. (**A**) The original training data (**D**) has m complex individuals and n characteristics (so it is m x n). (**B**) There are three various machine learning models (GBRT, AdaR, BagR) before training on D. (**C**) All model gives a forecast of the event, which is then cast into the second level of training data (Dnew), which is now m × 3. That is, three forecasts become characteristics of the second layer machine learning model (s). (**D**) The characteristics are trained on the second level model (XGBR) to produce the ultimate outcome.

Among machine learning methods, the performance of ensemble learning methods^56–62^ is very superior, so we use ensemble learning methods to predict the binding affinity of protein-DNA complexes. As one of the unique ensemble learning algorithms of ensemble learning, the stacking heterogeneous ensemble approach has a superior appearance. The flowchart of our method is displayed in Fig. 6. In our method, the stacking heterogeneous ensemble model includes two layers and contains one or more machine learning models in each layer. As shown in Fig. 6, there are three conventional machine learning models on the first layer of the PreDBA method, including the Gradient Boosted Regression Tree model, the Adaboost Regression model, and the Bagging Regression model. And there is a single one machine learning model, XGBoost Regression model, in the second layer.
